# An Improved Sensorless Hybrid Control Method of Permanent Magnet Synchronous Motor Based on I/F Startup

**DOI:** 10.3390/s23020635

**Published:** 2023-01-05

**Authors:** Bowen Ning, Yiheng Zhao, Shimin Cheng

**Affiliations:** Engineering Research Center for Metallurgical Automation and Measurement Technology of Ministry of Education, Wuhan University of Science and Technology, Wuhan 430081, China

**Keywords:** permanent magnet synchronous motor (PMSM), speed-sensorless control system, I/F startup, extended Kalman filter (EKF), hybrid control, power angle estimation

## Abstract

To realize permanent magnet synchronous motor (PMSM) in the full speed domain without speed sensor operation, a hybrid control method combining I/F startup and extended Kalman filter (EKF) is proposed in this paper. This method employs I/F startup to transition at low speed, effectively resolving the issue that the position estimation method based on the back electromotive force (EMF) model fails at zero speed and low speed, and converts to EKF for speed closed-loop vector control at medium and high speed. Moreover, a new feedback regulation mechanism as a solution to the problem of smooth switching between the two methods is proposed. First, the power angle is determined based on the relationship between the given I/F frequency and the estimated EKF position angle. Using the information of power angle, the damping torque of the system is increased to reduce velocity fluctuations during I/F startup. In addition, the balance point of current and position error angle is adjusted using the closed-loop information of position error angle to reduce the torque abrupt change before and after switching, thereby making the motor switching process to EKF speed closed-loop control more stable. Finally, simulation results are used to verify the effectiveness of the proposed scheme.

## 1. Introduction

Permanent magnet synchronous motors (PMSM) have been widely used in industrial applications and electric vehicles due to their high power density, small size, and low weight [1,2]. In certain application fields, such as fans, pumps, compressors, and home appliances, the dynamic performance requirements of motors are not as stringent as in the automotive and robotics industries. However, industrial costs and production environment usually limit the use of position sensors in these applications. Therefore, it is necessary to study a reliable and stable position sensorless PMSM control system [3].

Current sensorless position control methods are primarily divided into two categories based on the injection of high-frequency signals and the estimation of back electromotive force (EMF) [4]. Methods for injecting high-frequency signals include the rotating high-frequency signal injection method [5], the pulse vibration high-frequency signal injection method [6], high-frequency square wave signal injection method [7], etc. By injecting a specific high-frequency voltage into the stator windings, these techniques, which typically exploit the salient pole characteristic of the motor, could extract the signal carrying position information from the current. The signal injection method with a high frequency yields superior performance characteristics at zero and low speeds [8]. However, due to its reliance on the salient polarity of the motor, this method often fails for surface permanent magnet synchronous motors (SPMSM) with weak salient polarity. The estimation methods based on back EMF primarily consist of a model reference adaptive system [9], sliding mode observer [10], extended Kalman filter observer [11], etc. These methods obtain rotor position data using the back-EMF mathematical model of the motor, which is better suited for providing precise estimates at medium and high speeds [12]. However, due to the smaller back EMF at low speeds, such methods usually fail at zero and low speeds. To realize the starting process of a motor at zero speed and low speed, it is therefore possible to choose an open-loop startup strategy regardless of rotor position.

V/F (constant voltage–frequency ratio) control is a common open-loop startup strategy for induction motor control [13]. Although the V/F control method has a simple structure, it is unstable and inefficient [14]. To address these issues, a torque compensation method based on instantaneous power measurement is proposed to enhance the control performance of the V/F method [15,16]. However, the actual control performance depends on the parameters chosen. The I/F control method is another open-loop startup method that is more suitable for sensorless PMSM vector control [17,18]. During the start-up phase of this method, a constant current vector and a ramped speed command are given, and the integrated value of the speed command is used as the rotor’s reference position. I/F startup has the control characteristics of closed-loop current and open-loop speed. Moreover, it has a low reliance on motor parameters. Therefore, the control system’s stability can be enhanced. However, since the given current is determined by the load, excessive current will result in poor I/F control efficiency. Meanwhile, it easily can become out-of-step if the given current is too small.

Based on the aforementioned content and analysis, this paper proposes a hybrid control strategy, which utilizes the I/F startup method for low-speed transition, to address the deficiencies of the EMF model at zero and low speed, and the large speed fluctuations and low control efficiency of I/F startup [19]. When the speed reaches a predetermined threshold, it switches to medium-high sensorless vector control based on the position observer of the back electromotive force, which has a better effect and achieves sensorless control in the full-speed domain. Some position observers, such as the sliding mode observer, have better dynamic response due to their switching characteristics, but also bring certain chattering for observation angle. The chattering phenomenon will cause the observation angle to become unsmooth, thus increasing the instability of the whole observation system; therefore, it is necessary to design a filter to process the angle signal additionally. In contrast, the extended Kalman filter has a certain filtering effect on system noise, so its observation angle is smoother. Thus, it is applied to the medium-high speed stage after the I/F startup. The I/F startup procedure is analyzed in depth, and a damping technique compensation method is proposed to stabilize the I/F startup speed. In addition, a feedback regulator is designed to automatically adjust the position angle difference between the rotor reference position given by the I/F startup and the observation position information given by the position observer to improve the stability of the conversion process.

The remainder of this paper is organized as follows. Section 2.1 describes the mathematical model of the PMSM based on the back EMF and develops the extended Kalman filter method. In Section 2.2, the I/F startup strategy is discussed, focusing on the characteristics of the acceleration and uniform speed stages. Section 2.3 presents an enhanced method for combining I/F startup with the extended Kalman filter. The angle information estimated by the Kalman filter is used to adjust the given I/F angle, and specific solutions to the problems of I/F speed fluctuation and low control efficiency are provided. Section 3 discusses the performance of the proposed startup strategy, and Section 4 concludes the research presented in this paper.

## 2. Materials and Methods

### 2.1. PMSM Mathematical Model and EKF

The principle of sensorless motor control varies for different motor models. In general, a mathematical model in a rotating coordinate system is used to obtain first the motor speed and then the motor’s rotor position through integration [20]. The method proposed in this paper has strict requirements for the rotor position, so a mathematical model in a static coordinate system is used to obtain the rotor position. The motor speed is derived by differentiating the rotor position.

For mathematical modeling of PMSM, the extended back electromotive force model proposed in the literature [21] is utilized, and its current formula in the static coordinate system is as follows:

(1) $$\left\{ \begin{array}{l} \frac{di_{\alpha }}{dt}=\frac{1}{L_{d}}\left[-Ri_{\alpha }-\omega_{e}\left(L_{d}-L_{q}\right)i_{\beta }+E_{X}\sin\theta +u_{\alpha }\right]\\ \frac{di_{\beta }}{dt}=\frac{1}{L_{d}}\left[-Ri_{\beta }+\omega_{e}\left(L_{d}-L_{q}\right)i_{\alpha }-E_{X}\cos\theta +u_{\beta }\right] \end{array}\right.$$

where $i_{\alpha }$, $i_{\beta }$, $u_{\alpha }$, and $u_{\beta }$ are the stator current and stator voltage in the static coordinate system. R represents the stator phase resistance; $L_{d}$ and $L_{q}$ are the motor d-axis and q-axis inductances, respectively. θ represents the electrical angular position of the rotor, $\omega_{e}$ denotes the rotor electrical angular velocity, $\psi_{f}$ represents the permanent magnet flux linkage, and $E_{X}$ represents the amplitude of the extended back EMF, which is expressed as follows:

(2) $$E_{X}=\left(L_{d}-L_{q}\right)\left(\omega_{e}i_{d}-\frac{di_{q}}{dt}\right)+\omega_{e}\psi_{f}$$

The estimated rotor position is:

(3) $$\theta =\arctan\left(\frac{-E_{X}\sin\theta }{E_{X}\cos\theta }\right).$$

Let $E_{X}\sin\theta$ be $E_{\alpha }$, $E_{X}\cos\theta$ be $E_{\beta }$. Select state variable, input variable, and output variable as $x={\left[\begin{array}{cccc} i_{\alpha } & i_{\beta } & E_{\alpha } & E_{\beta } \end{array}\right]}^{T}$, $u={\left[\begin{array}{cc} u_{\alpha } & u_{\beta } \end{array}\right]}^{T}$, and $y={\left[\begin{array}{cc} i_{\alpha } & i_{\beta } \end{array}\right]}^{T}$. Add system noise and measurement noise, and discretize them:

(4) $$\{\begin{array}{c} x_{k+1}=f\left(x_{k},u_{k}\right)+w_{k}\\ y_{k}=H_{d}x_{k}+v_{k} \end{array},$$

where $H_{d}$ is the output matrix and f represents the nonlinear function after discretization, which is expressed as:

$$f=\left[\begin{array}{c} i_{\alpha ,k}+\frac{T_{s}\left[-Ri_{\alpha ,k}-\omega_{e,k}\left(L_{d}-L_{q}\right)i_{\beta ,k}+u_{\alpha ,k}-E_{\alpha ,k}\right]}{L_{d}}\\ i_{\beta ,k}+\frac{T_{s}\left[-Ri_{\beta ,k}+\omega_{e,k}\left(L_{d}-L_{q}\right)i_{\alpha ,k}+u_{\beta ,k}-E_{\beta ,k}\right]}{L_{d}}\\ E_{\alpha ,k}-T_{s}\omega_{e,k}E_{\beta ,k}+W_{1}\\ E_{\beta ,k}-T_{s}\omega_{e,k}E_{\alpha ,k}+W_{2} \end{array}\right].$$

The research object of this paper is to design a control mode of surface permanent magnet synchronous motor (SPMSM). For SPMSM, $L_{d}=L_{q}$. $W_{1}$ and $W_{2}$ are transient high-frequency components, which can be ignored when calculating the extended back EMF. $w_{k}$ and $v_{k}$ represent the system noise and measurement noise, respectively. In practice, these two noises are random and independent of each other, and conform to a normal distribution with an expectation value of zero. Their covariance matrices Q and R are described as follows:

(5) $$\begin{array}{l} \mathrm{cov}\left(w\right)=E\left[w,w^{T}\right]=Q\\ \mathrm{cov}\left(v\right)=E\left[v,v^{T}\right]=R \end{array}.$$

EKF, which is an extended application of the Kalman filter algorithm in a nonlinear system, is a type of optimal recursion method. It expands the current working point of the nonlinear function through Taylor series expansion and ignores the higher-order terms; then the system is approximated to a linear system after linearization. Aiming for minimum error covariance, EKF could obtain the optimal estimation result through prediction step and update step in a noisy system. The steps of EKF for the permanent magnet synchronous motor system are as follows [22]:

(1) State prediction. Predict the next moment’s state vector based on the current input and the previous state estimate.

(6) $${\tilde{x}}_{k+1}=f\left({\tilde{x}}_{k},u_{k}\right),$$

where $T_{s}$ represents the sampling period, and ${\hat{x}}_{k}$ and ${\hat{x}}_{k+1}$ represent the current state and the estimated value of the state at the next moment, respectively.

(2) Calculate the output ${\tilde{y}}_{k+1}$ at the next moment:

(7) $${\tilde{y}}_{k+1}=H_{d}{\tilde{x}}_{k+1}.$$

(3) Calculate the error covariance matrix:

(8) $${\tilde{p}}_{k+1}={\hat{p}}_{k}+T_{s}\left(F_{k}{\hat{p}}_{k}+{\hat{p}}_{k}F_{k}^{T}\right)+Q,$$

where $F_{k}$ represents the Jacobian matrix.

(4) Calculate the gain matrix:

(9) $$K_{k+1}={\tilde{p}}_{k+1}H_{d}^{T}{\left(H_{d}{\tilde{p}}_{k+1}H_{d}^{T}+R\right)}^{-1}.$$

(5) State estimation:

(10) $${\hat{x}}_{k+1}={\tilde{x}}_{k+1}+K_{k+1}\left(y_{k+1}-{\tilde{y}}_{k+1}\right).$$

(6) Update the error covariance matrix:

(11) $${\hat{p}}_{k+1}={\tilde{p}}_{k+1}-K_{k+1}H_{d}{\tilde{p}}_{k+1},$$

where ${\hat{p}}_{k+1}$ represents the covariance matrix used in the next state estimation.

### 2.2. Current Closed-Loop Speed Open-Loop I/F Startup Analysis

**Figure 1:**
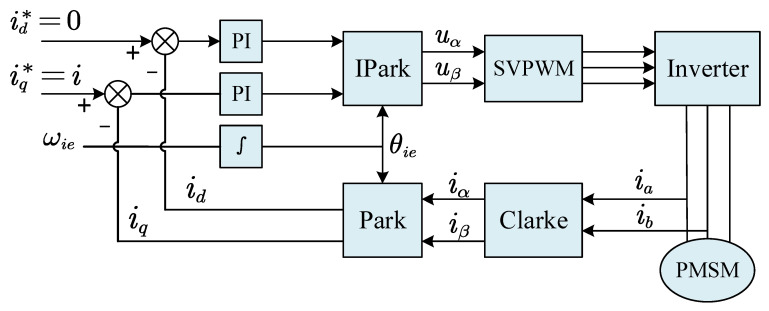
I/F startup system block diagram.

This paper uses the I/F startup strategy to transition the motor from zero to medium-high speed. I/F startup requires a proper q-axis current amplitude and current rotation frequency. Typically, the precise value is determined offline based on the load, and the d-axis current is set to zero. Figure 1 depicts the block diagram of the PMSM speed open-loop I/F startup system [17].

**Figure 2:**
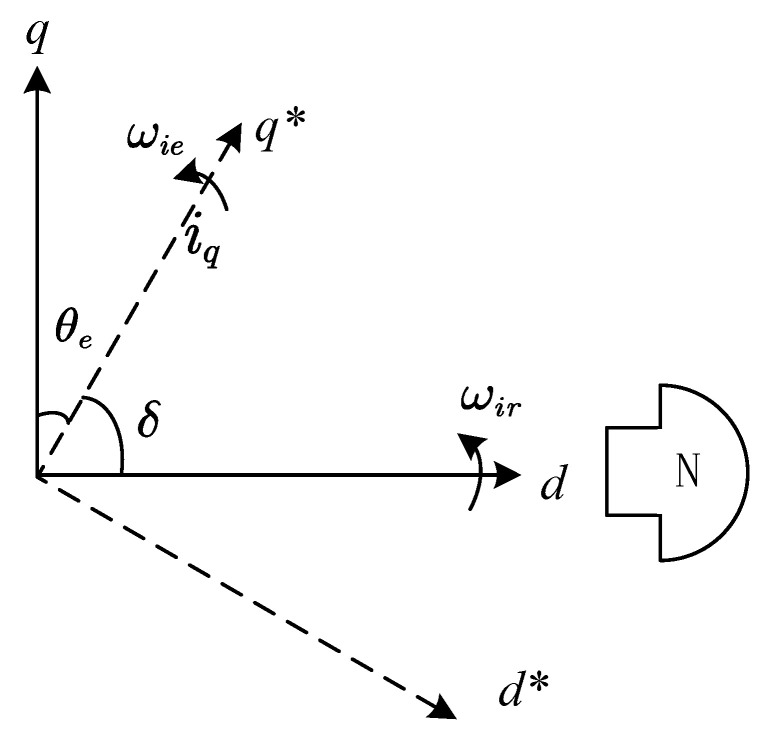
The rotating *d-q* axis and the virtual d*-q* axis.

The mechanical formula of SPMSM can be expressed as:

(12) $$J\frac{d\omega_{r}}{dt}=\frac{3}{2}n_{p}\psi_{f}i\sin\delta -B\omega_{r}-T_{L},$$

where $\omega_{r}$ denotes the mechanical angular velocity of the motor, $n_{p}$ represents the number of pole pairs of the motor, and *i* is the magnitude of the current vector. δ represents the angle between the current vector and the permanent magnet excitation flux linkage, which is called the power angle later. J represents the moment of inertia, B denotes the damping coefficient, and $T_{L}$ is the load torque. For a clearer representation of the relationship between a given current vector and rotor position, the virtual rotating coordinate systems d* and q* are used to represent the *d* and *q* axes of the given current vector. The electric angle of axis *d* in the virtual coordinate system where the current vector located is $\theta_{ie}$, the rotational electric angular velocity is $\omega_{ie}$, and the rotating electric angular velocity of the coordinate system where the rotor is located is $\omega_{ir}$, as shown in Figure 2.

Denote the electrical angle velocity of the given current vector as $\omega_{ie}$ and the given mechanical angular velocity as $\omega_{e}=\frac{\omega_{ie}}{n_{p}}$. Then, the difference between the given mechanical angular velocity and the mechanical angular velocity of the rotor is denoted by Δω. This leads to the following formula:

(13) $$\delta^{\prime }=n_{p}\Delta \omega =n_{p}\left(\omega_{e}-\omega_{r}\right).$$

Substitute Formula (13) into (12) to obtain the differential formula:

(14) $$J\frac{\delta^{\prime \prime }}{n_{p}}+B\frac{\delta^{\prime }}{n_{p}}+\frac{3}{2}n_{p}\psi_{f}i\sin\delta =J{\omega^{\prime }}_{e}+B\omega_{e}+T_{L}.$$

When the above formula reaches a steady-state, $\omega_{e}$ changes very slowly. Thus, it can be considered a constant value. As $\delta^{\prime }$ converges to zero, the steady-state condition is:

(15) $$sign\left[\delta^{\prime }\right]\cdot sign\left[{\left(\sin\delta \right)}^{\prime }\right]>0.$$

Therefore, the conditions that are satisfied when the PMSM open-loop I/F startup mode is stable in operation are:

(16) $$-\frac{\pi }{2}<\delta <\frac{\pi }{2}.$$

Define the angle between the given current vector angle and the rotor q-axis as the error angle $\theta_{e}$:

(17) $$\theta_{e}=\frac{\pi }{2}-\delta .$$

in the control mode with *i_d_* = 0 and *i_q_* = *i*. According to Formula (12), the power angle relationship under stable conditions is obtained as:

(18) $$\frac{3}{2}n_{p}i_{q}\psi_{f}\sin\delta =B\omega_{ie}+T_{L}.$$

Solve the stable point of the power angle:

(19) $$\delta_{0}=\arcsin\left(\frac{B\omega_{e}+T_{L}}{\frac{3}{2}n_{p}i_{q}\psi_{f}}\right).$$

According to Formula (17), the error angle of the stable point is:

(20) $$\theta_{0}=\arccos\left(\frac{B\omega_{e}+T_{L}}{\frac{3}{2}n_{p}i_{q}\psi_{f}}\right).$$

**Figure 3:**
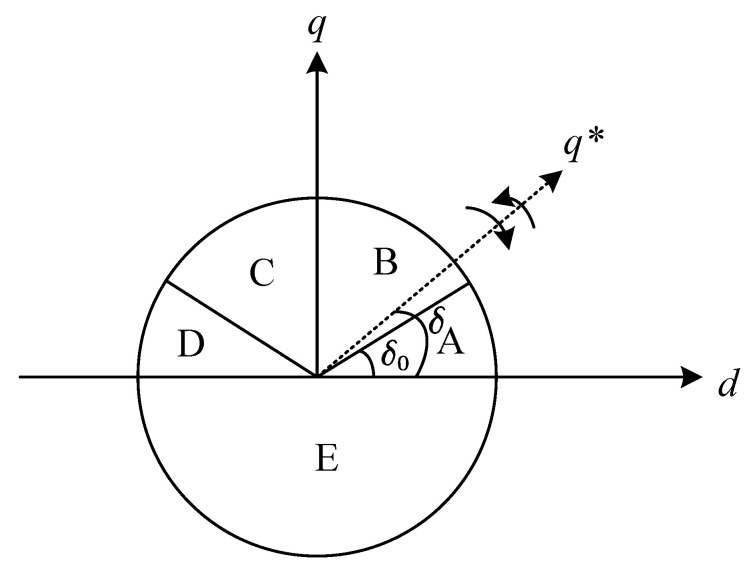
The virtual coordinate system and the real coordinate system with error angle regions.

According to the above derivation and analysis, during the process of speed open-loop I/F startup, by selecting the appropriate given current vector value and current rotation frequency, the rotor of the motor can rotate with the given current frequency and maintain dynamic balance after the speed is stable, and the power angle δ converges to $\delta_{0}$. As shown in Figure 3, counterclockwise rotation is taken as the positive direction. Considering that the load and friction force are always positive, when the power angle reaches $\delta_{0}$, the resultant force with the motor torque is zero. Therefore, $\delta_{0}$ is the power angle balance point. When the power angle δ moves to the B region or the C region, the motor accelerates and moves into the A region. In region D, the motor decelerates to move, and when it enters region E, a torque opposite to the direction of motor rotation is generated.

During the operation of the motor, the torque generated by the motor must be initially positive, that is, δ is positive, to ensure that the motor can overcome the load torque and drive the motor to rotate. When the motor speed is stable, the torque generated by the motor and the load torque are balanced. In Figure 3, the torque balance point is at the intersection of the AB and CD regions. When δ is in the C region and increases, the torque will decrease, which will cause δ to increase further and eventually become out-of-step. Thus, if the given positive direction is counterclockwise, only when $\delta_{0}$ is 0 to $\frac{\pi }{2}$ is the operating range of the motor I/F startup stable, that is, the AB area in the figure.

On the other hand, the actual speed of the motor constantly fluctuates around the specified value, and there are acceleration and deceleration processes during the startup process. Therefore, the overall process of the motor is a process of variable acceleration movement. δ also moves back and forth around the power angle balance point $\delta_{0}$ and gradually converges. The convergence rate is dependent on the damping coefficient. When the load damping is low, the motor speed converges slowly to its steady-state value. The final steady-state equilibrium point $\delta_{0}$ has a non-linear positive correlation with the current *i_q_* in the stable operation range under the condition that the rotational speed torque and the motor constant remain unchanged.

Since the I/F startup is an open-loop control mode, the initial position of the motor rotor is unknown, and the starting speed cannot precisely match the specified value. With the I/F startup, the initial position of the motor must be known. Suppose the initial given position is too different from the actual motor position. In that case, an opposite torque will be generated, causing the I/F startup to not be a strict uniform acceleration start, which will impact the motor rotor speed’s follow-up effect. Currently, the prepositioning method is often used, in which a constant current vector is provided, and the generated torque drags the motor rotor to the predetermined position. However, there is a situation where the difference between the given current vector and the rotor position is at a π angle. The lower motor torque is zero, resulting in positioning failure. In practice, a slow rotating current vector is frequently provided to guarantee the efficacy of the prepositioning method.

### 2.3. Smooth Switching between IF Control and EKF

**Figure 4:**
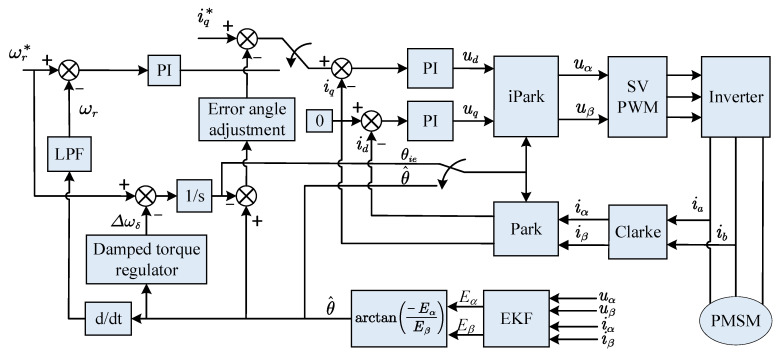
I/F startup and EKF sensorless hybrid control scheme block diagram.

After the I/F startup, when the motor speed reaches a certain value, the position $\hat{\theta }$ estimated by the EKF based on the motor back-EMF model is valid. At this time, the I/F startup can be adjusted based on the estimated position of the EKF. The previous analysis demonstrates that the I/F startup strategy has a problem with fluctuating speed. The mismatch between the power angle and current value during stability will result in low current utilization. In this paper, a new method is proposed for adjusting the electrical angle frequency and current given by I/F startup using the effective position information estimated by EKF to decrease the speed fluctuation and increase the current utilization rate. Hence, a smooth transition to EKF speed closed-loop control is possible through the adjustment action. The block diagram of the whole control process of starting and switching is shown in Figure 4.

The speed fluctuation can be reduced by adding the damping torque component. The method requires that the power angle information is obtained from the position information estimated by the EKF and the information provided by the I/F startup. The final convergence power is determined using the fluctuation information of the estimated power angle. The value of the angle $\delta_{0}$ is compared to the current actual power angle, and the speed fluctuation is suppressed by the input of this value. This method effectively reduces speed fluctuations throughout the entire starting procedure and accelerates the convergence speed of the power angle.

The power angle is obtained from the angle estimated by EKF and the angle given by I/F startup as the following:

(21) $$\delta =\frac{\pi }{2}+\theta_{ie}-\hat{\theta }.$$

It can be seen from the previous analysis that in the I/F startup, the actual power angle δ reciprocates around the steady-state power angle $\delta_{0}$. The angle between them is:

(22) $$\Delta \delta =\delta -\delta_{0}.$$

When $\delta >\delta_{0}$ and $\delta^{\prime }=0$, record the value of the power angle at this moment as $\delta_{\max}$. When $\delta <\delta_{0}$ and $\delta^{\prime }=0$, record the value of the power angle at this moment as $\delta_{\min}$. From Formula (19), it can be seen that the stable power angle $\delta_{0}$ is related to the speed and the load. When the torque load is constant, only the speed affects the power angle. It can be considered that the stable point $\delta_{0}$ of the power angle also changes uniformly with time in the stage of uniform acceleration. When the speed is at a constant speed, the stable point $\delta_{0}$ of the power angle remains unchanged.

**Figure 5:**
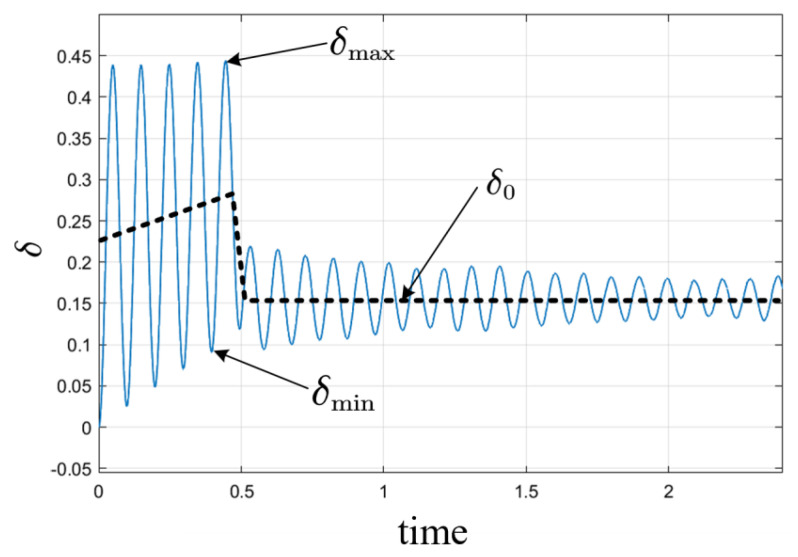
I/F startup actual power angle and stable power angle.

As shown in Figure 5, the steady-state power angle that can be estimated when the speed fluctuation reaches its extreme value is approximately:

(23) $${\hat{\delta }}_{0,m+n}=\frac{\delta_{\max,m}+\delta_{\min,n}}{2},$$

where $\delta_{\max,m}$ is the *m*-th maximum time, $\delta_{\max,n}$ is the *n*-th minimum time, and ${\hat{\delta }}_{0,m+n}$ is the (*m* + *n*)-th extreme time, (*m*, *n* = 0, 1, 2…). In each cycle of speed fluctuation when the motor is accelerating uniformly, the power angle at other times of the speed fluctuation extreme value can be calculated by using the information of the power angle at the previous two extreme values as follows:

(24) $$\delta_{0}={\hat{\delta }}_{0,m+n}+\Delta t*b.$$

The power angle at the last extreme moment is denoted by ${\hat{\delta }}_{0,m+n-1}$, and Δt is the time interval between the ${\hat{\delta }}_{0,m+n}$ moment and the ${\hat{\delta }}_{0,m+n-1}$ moment. b represents the proportional coefficient and its expression is:

(25) $$b=\frac{{\hat{\delta }}_{0,m+n}-{\hat{\delta }}_{0,m+n-1}}{\Delta T},$$

where ΔT represents the time difference between the first two extreme moments. When the speed is stable, b=0. After identifying the current power angle, the formula for adding a given damping torque to a given speed is as follows:

(26) $$\Delta \omega_{\delta }=-h*\Delta \delta ,$$

where *h* is the feedback coefficient.

**Figure 6:**
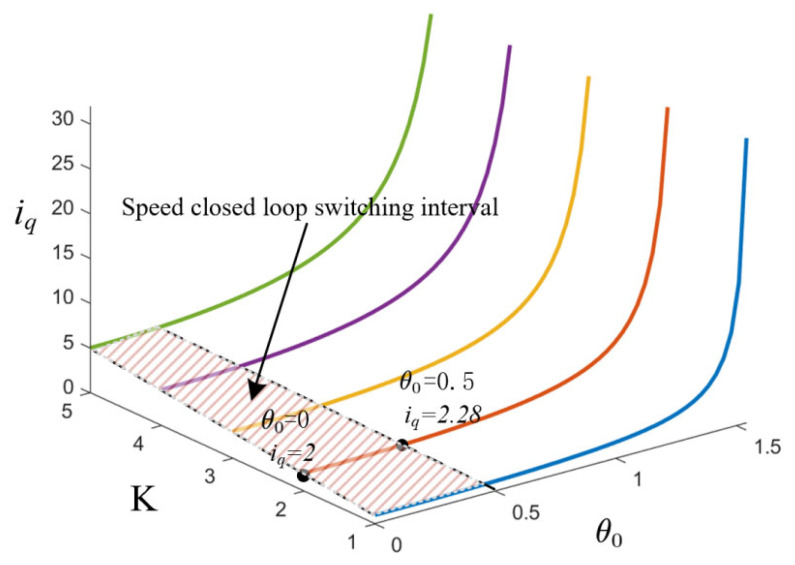
The relationship between the error angle and the current under different K values when the speed is stable.

Adjusting the given current vector amplitude *i_q_* can change the steady-state position of the power angle. To achieve a smooth transition between the two control methods, it is necessary to adjust the given current *i_q_* after the speed has been stabilized so that the virtual coordinate system in which the given current resides is identical to the one. Through the stable error angle of $\theta_{0}$ determined above, the current is feedback adjusted to increase the utilization rate of the current. For the SPMSM, the ideal situation is when the $\theta_{0}$ converges to zero. However, when $\theta_{0}=0$, the stability of the system will be greatly reduced. According to Formula (20), the relationship between the current and the error angle in the steady-state of the speed can be obtained as follows:

(27) $$i_{q}=\frac{K}{\cos\theta_{0}},$$

where $K=\frac{2(B\omega_{e}+T_{L})}{3n_{p}\psi_{f}}$. When the speed and load are constant, K is constant too. As a result, the balance point curve of the *i_q_* and stable error angle under different loads and speeds can be obtained, as shown in Figure 6.

The figure illustrates that when the load is constant, and the rotational speed is stable, the sensitivity of the change in the difference angle to the current is greatly reduced as the difference angle approaches zero. This will result in the entire system becoming unstable. This paper designs a regulation mechanism to rapidly reduce the feedback amount to the current when the difference angle is less than a certain value, and to change the position of the switching point to increase the overall system’s stability during the current regulation process. This ensures that the current remains constant before and after switching to EKF high-speed control. Due to $\theta_{e}=\theta_{0}$ in steady state, the regulation mechanism is expressed as follows:

(28) $$\left\{ \begin{array}{l} i_{q}^{-}=K_{p}\theta_{e}+K_{i}\int e\left(\theta_{e}\right)d\theta_{e}\begin{array}{c} , \end{array}\sigma <\theta_{e}<\frac{\pi }{2}\\ i_{q}^{-}={\left(\frac{\sigma -\theta_{e}}{\sigma }\right)}^{3}\left(K_{p}\theta_{e}+K_{i}\int e\left(\theta_{e}\right)d\theta_{e}\right)\begin{array}{c} , \end{array}\sigma <\theta_{e}<\frac{\pi }{2} \end{array}\right.,$$

where *K_p_* and *K_i_* represent PI regulator parameters, $i_{q}^{-}$ denotes the reduction value of the given current *i_q_*, and σ is the switching point of the set interval. When the error angle $\theta_{e}$ is reduced to σ, it enters the switching interval. The feedback regulation relation between given current *i_q_* and error angle is changed, which can reduce the change of $\theta_{e}$ to adjust the current. The control mode under EKF can be regarded as $\theta_{e}=0$ during closed-loop operation after switching. As can be seen from Figure 6, the current *i_q_* can be calculated as 114% of the speed closed-loop when $\theta_{e}=0.5$. Thus, at $\theta_{e}$ less than the set point σ, the current *i_q_* at this time and the *i_q_* after switching to the speed closed loop can be considered to be consistent. However, the stability is better than when $\theta_{e}=0$. Adjusting the PI regulator requires the addition of a low-pass filter because the switching current feedback is not smooth, and signal interference will result in a sudden change in torque and unstable rotation speed.

## 3. Simulation Results and Analysis

To verify the effectiveness of the proposed scheme, a simulation model from I/F startup to EKF speed closed-loop control was developed in a Matlab/Simulink environment. The simulation parameters for SPMSM are shown in Table 1. The DC bus voltage *V_dc_* = 311 V, and the controller the current loop control frequency *T_s_* = 10 KHz.

**Figure 7:**
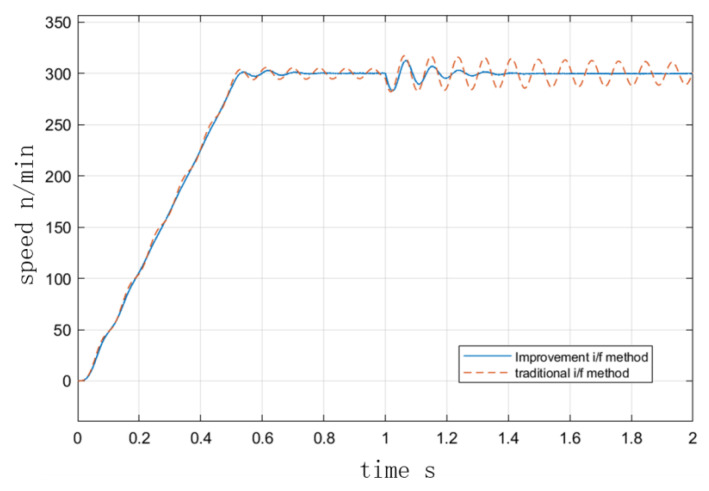
I/F startup with and without torque damping at no load.

**Figure 8:**
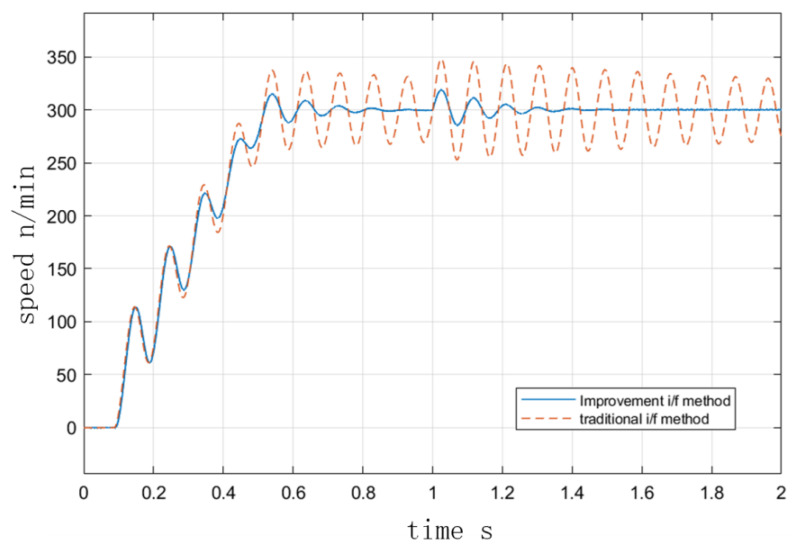
I/F startup with and without torque damping under load.

**Figure 9:**
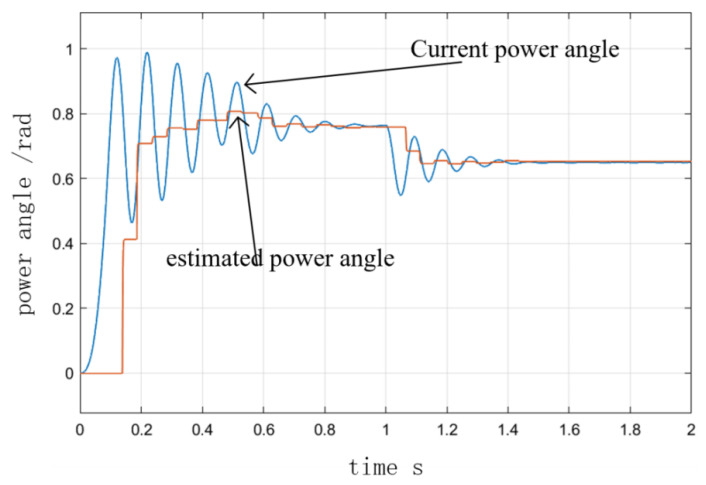
Current power angle and estimated steady-state power angle response.

Figure 7 and Figure 8 depict the velocity curves with and without damping torque feedback at the I/F startup. In the simulation, the speed was set to accelerate uniformly to 300 r/min within 0.5 s and to maintain that speed after 0.5 s. As depicted in Figure 7, the initial given current was 10 A, the initial state of the motor was no-load, and the load increased to 1 N·m at 1 s. In Figure 8, the initial condition of the motor was 4 N·m load. Figure 8 depicts the operation curve when the load decreased to 3 N at 1 s. It can be seen that after the initial fluctuation period, the torque fluctuation in the I/F startup process was quickly suppressed due to the presence of torque damping, and the speed could quickly recover to stability after the change of torque, indicating that the improved method improves the stability performance of I/F startup. Figure 9 illustrates the curves of the actual and predicted steady-state power angle during I/F startup.

**Figure 10:**
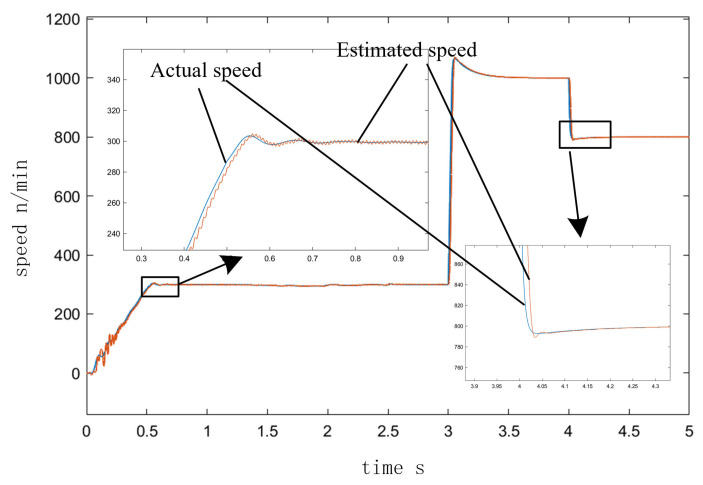
I/F startup to EKF speed closed-loop speed response.

**Figure 11:**
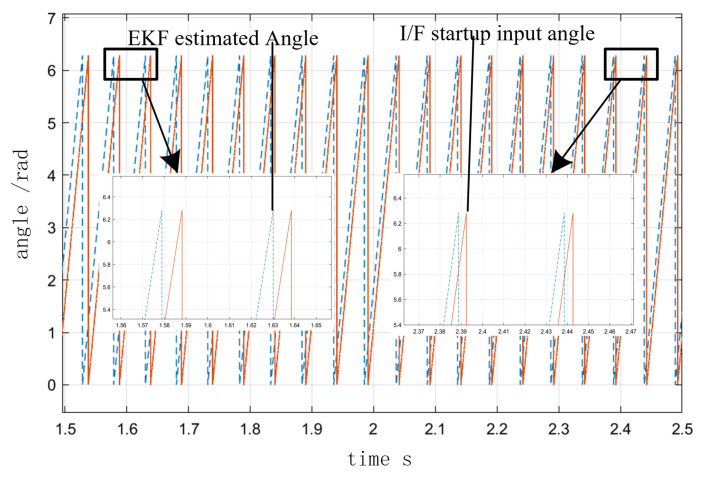
EKF estimated position and I/F startup given position angle during automatic adjustment of I/F startup current.

**Figure 12:**
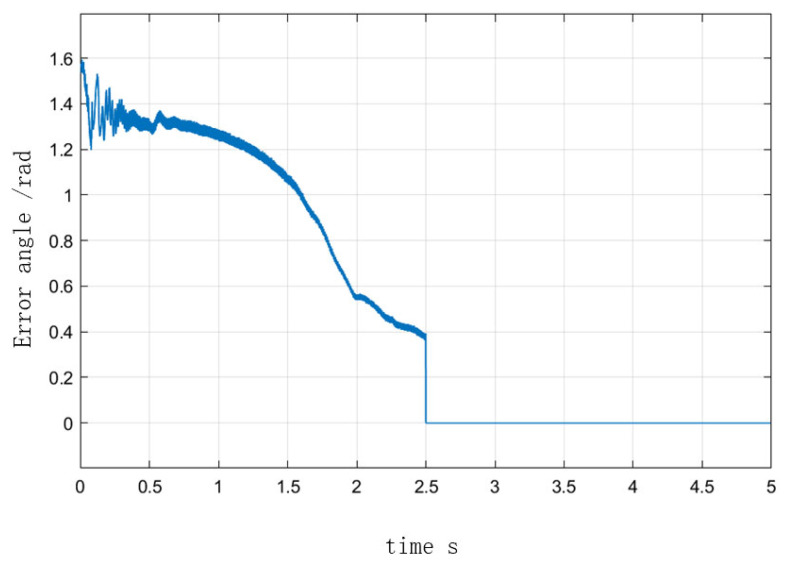
Error angle response from I/F startup to EKF speed closed-loop.

**Figure 13:**
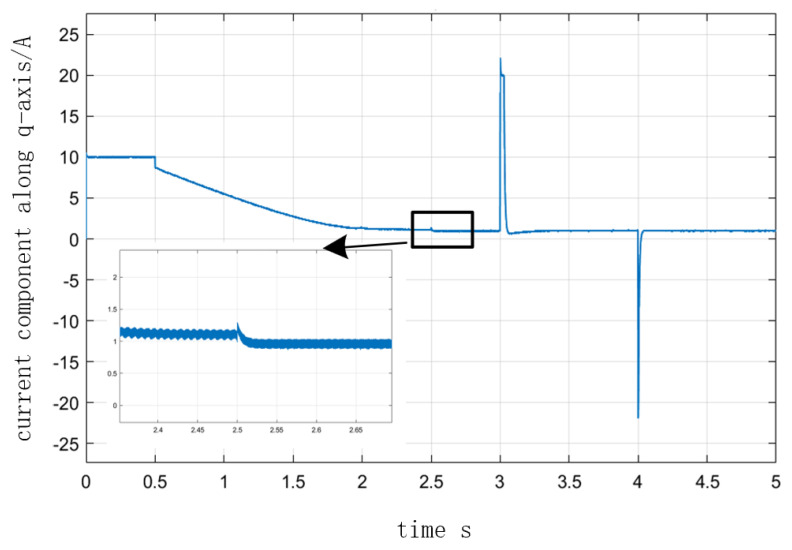
The q-axis current response from I/F startup to EKF speed closed-loop.

Figure 10 depicts the speed curve of the entire process from I/F startup to EKF speed closed-loop operation. In the simulation of a 2 N·m load on a given motor, the speed was set to accelerate uniformly to 300 r/min in 0.5 s and to maintain that speed after 0.5 s. At 2.5 s, the command to switch to the position observed by EKF was issued, and speed feedback was added. The speed was increased to 1000 r/min at 3 s and decreased to 800 r/min at 4 s, and the value of σ was 0.5 in the adjustment process. Figure 11 illustrates the angle curves of the estimated position of EKF and the given position of I/F startup during the automatic adjustment of the I/F startup current. When the I/F startup speed reached a constant speed between 0.5 s and 2.5 s, Figure 12 depicts the change in the difference angle during current regulation. The current *i_q_* curve throughout the entire process is depicted in Figure 13.

After combining the improvement methods of closed-loop adjustment, as shown in Figure 11 and Figure 12, the current and differential angle were automatically adjusted by the feedback effect to a new equilibrium state, with the current reaching the switching surface and the degree of difference decreasing as the system maintained a steady state until the switch to EKF speed closed-loop control. As can be seen in Figure 10 and Figure 13, the value of the current *i_q_* in 2.5 s was fundamentally consistent, before and after the switch to the speed closed-loop and speed fluctuations were small. Meanwhile after the switch process was completed, the closed-loop sensorless control based on EKF had a good response. According to the above analysis, it can be seen that the proposed method achieves the reliable transition from zero speed and low speed to medium-high speed, and avoids the shortage of low speed of EKF, which proves the feasibility of the improved method in this paper.

## 4. Results

When a sensorless control scheme based on back electromotive force model is implemented for SPMSM, it often fails at zero and low speeds. Therefore, it is necessary to take the I/F startup for the transition from zero speed and low speed to high-speed switching. In this paper, a sensorless control based on EKF is adopted as the medium-high speed control scheme, and an improved method is proposed to reduce the speed fluctuation by increasing the torque damping in I/F startup and to solve the problem of smooth switching between the two methods. The proposed method is well-suited for startup processes at low speed and the transition to high speed of the sensorless control, which ensures a good robustness and adaptability of the whole control system.

## Figures and Tables

**Table 1:** SPMSM main parameters.

Parameter	Symbol	Value	Unit
Mechanical inertia	J	0.008	kg·m^2^
Damping coefficient	B	0.008	N·m·s
Stator resistance	$R_{s}$	2.875	Ω
Stator inductance	$L_{s}$	0.0085	H
Number of pole pairs	$n_{p}$	4	-
Rated torque	$T_{N}$	4	N·m
Rated speed	$n_{N}$	3000	r/min
Permanent-magnet flux	$\psi_{f}$	0.175	Wb

## Data Availability

The data presented in this study are available on request from the corresponding author. The data are not publicly available due to privacy.

## References

[1] Iqbal S., Habib S., Ali M., Shafiq A., Rehman A.U., Ahmed E.M., Khurshaid T., Kamel S. (2022). The Impact of V2G Charging/Discharging Strategy on the Microgrid Environment Considering Stochastic Methods. Sustainability.

[2] Iqbal S., Xin A., Jan M.U., Abdelbaky M.A., Rehman H.U., Salman S., Aurangzeb M., Rizvi S.A.A., Shah N.A. (2020). Improvement of Power Converters Performance by an Efficient Use of Dead Time Compensation Technique. Appl. Sci..

[3] Urbanski K., Janiszewski D. (2019). Sensorless Control of the Permanent Magnet Synchronous Motor. Sensors.

[4] Wang Y., Xu Y., Zou J. (2019). Sliding-Mode Sensorless Control of PMSM With Inverter Nonlinearity Compensation. IEEE Trans. Power Electron..

[5] Bai H. (2020). Position Estimation of a PMSM in an Electric Propulsion Ship System Based on High-Frequency Injection. Electronics.

[6] Wang S., Yang K., Chen K. (2019). An Improved Position-Sensorless Control Method at Low Speed for PMSM Based on High-Frequency Signal Injection into a Rotating Reference Frame. IEEE Access.

[7] Cui W., Zhang F.-X. (2018). A Novel Sensorless Rotor Position Estimation Method for PMSM Based on High-Frequency Square-Wave Voltage Injection with Less Iron Loss. Electr. Power Compon. Syst..

[8] Sun X., Cai F., Yang Z., Tian X. (2022). Finite Position Control of Interior Permanent Magnet Synchronous Motors at Low Speed. IEEE Trans. Power Electron..

[9] Sun X., Zhang Y., Tian X., Cao J., Zhu J. (2021). Speed Sensorless Control for IPMSMs Using a Modified MRAS With Gray Wolf Optimization Algorithm. IEEE Trans. Transp. Electrif..

[10] Sun X., Li T., Zhu Z., Lei G., Guo Y., Zhu J. (2021). Speed Sensorless Model Predictive Current Control Based on Finite Position Set for PMSHM Drives. IEEE Trans. Transp. Electrif..

[11] Tian L., Li Z., Wang Z., Sun X., Guo T., Zhang H. (2021). Speed-sensorless control of induction motors based on adaptive EKF. J. Power Electron..

[12] Shen J., Zhu Z., Howe D. (2002). Improved speed estimation in sensorless PM brushless AC drives. IEEE Trans. Ind. Appl..

[13] Perera P.C., Blaabjerg F., Pedersen J.K., Thogersen P. (2003). A sensorless, stable V/f control method for permanent-magnet synchronous motor drives. IEEE Trans. Ind. Appl..

[14] Sue S.-M., Hung T.-W., Liaw J.-H., Li Y.-F., Sun C.-Y. A new MTPA control strategy for sensorless V/f controlled PMSM drives. Proceedings of the 6th IEEE Conference on Industrial Electronics and Applications.

[15] Ancuti R., Boldea I., Andreescu G.-D. (2010). Sensorless V/f control of high-speed surface permanent magnet synchronous motor drives with two novel stabilising loops for fast dynamics and robustness. IET Electr. Power Appl..

[16] Agarlita S.-C., Coman C.-E., Andreescu G.-D., Boldea I. (2013). Stable V/f control system with controlled power factor angle for permanent magnet synchronous motor drives. IET Electr. Power Appl..

[17] Xing J., Qin Z., Lin C., Jiang X. (2020). Research on Startup Process for Sensorless Control of PMSMs Based on I-F Method Combined With an Adaptive Compensator. IEEE Access.

[18] Baratieri C.L., Pinheiro H. An I/F starting method for smooth and fast transition to sensorless control of BLDC motors. Proceedings of the Brazilian Power Electronics Conference.

[19] Shi H.-J., Nie X.-C. (2021). Composite control for disturbed direct-driven surface-mounted permanent magnet synchronous generator with model prediction strategy. Meas. Control.

[20] Consoli A., Scarcella G., Testa A. (2001). Industry application of zero-speed sensorless control techniques for PM synchronous motors. IEEE Trans. Ind. Appl..

[21] Chen Z., Tomita M., Doki S., Okuma S. (2003). An extended electromotive force model for sensorless control of interior permanent-magnet synchronous motors. IEEE Trans. Ind. Electron..

[22] Smidl V., Peroutka Z. (2011). Advantages of Square-Root Extended Kalman Filter for Sensorless Control of AC Drives. IEEE Trans. Ind. Electron..

